# Metropolitan Hospital Sunday Fund Procedure and Its Amendment

**Published:** 1911-12-30

**Authors:** 


### METROPOLITAN HOSPITAL SUNDAY FUND PROCEDURE AND ITS AMENDMENT.

We give elsewhere a full report of the discussions at the annual meeting of the Metropolitan Hospital Sunday Fund, held in the Egyptian Hall of the Mansion House on December 21. The main theme of the discussion was the procedure of the authorities of St. George's Hospital, the rules laid down by the King's Hospital Fund, and the course adopted by the Distribution Committee of the Sunday Fund. These matters the Lord Mayor, Sir Thomas Crosbie, who fortunately is a member of the medical profession, has pledged himself to investigate thoroughly. This renders it undesirable to discuss the principles involved here, nor is it necessary because the position was made clear in our issue of December 23, page 311, in which the facts are set forth by the Treasurer of St. George's Hospital, and in the report of the Distribution Committee of the King's Fund.

Quite apart from the subject-matter of the discussion on December 21 we welcome it as evidence that there is life and force and future prosperity in the Metropolitan Hospital Sunday Fund movement, if its Council and Committees show statesmanship and wisdom in handling the questions which have arisen mainly on matters of procedure at several successive annual meetings of the Fund. Those who are interested in the question should refer back to the full reports of the proceedings at the annual meeting of the Metropolitan Hospital Sunday Fund as published in The Hospital of December 21, 1907, page 327; December 26, 1908, page 337; December 25, 1909, page 369; December 24, 1910, page 391. Such a reference will show that, owing to the absence of clear rules of procedure for the conduct of the business of the annual meeting of the Metropolitan Hospital Sunday Fund, the constituents have become increasingly restive, because a general impression has been created in the minds of the public, through the constituents of the Fund, that the constitution as at present drawn and administered does not give the contributing congregations and their representatives the necessary facilities impartially and fully to state their views and wishes in regard to the distribution of the funds collected. To state tersely this fact is to reveal at once the main causes which have contributed to diminish during recent years the aggregate sum raised by congregational collections in London for the voluntary hospitals. Hospital Sunday must depend for its success upon the conviction and earnest purposefulness of the clergy and ministers of all denominations who advocate its claims, and upon an assured feeling on the part of their congregations and the public generally that the raising of money does confer the privilege of having a voice not only in the management of the Fund itself, but in the distribution of the funds which are raised for the hospitals by the churches.

The mere fact that the annual meeting is held each year under the presidency of the Lord Mayor in the Egyptian Hall of the Mansion House necessitates that the meeting shall be so conducted as to give the fullest and fairest opportunity to every accredited representative to take part in the discussions. To hamper or render impotent independent speakers by raising mere technicalities in order to shorten the proceedings and confine them as much as possible to the routine resolutions submitted on the authority of the Council would prove a fatal policy. Our memory goes back to the time before the Fund had a constitution, when the late Sir Morrell Mackenzie filled the Mansion House at one such meeting with the supporters and out-patients of his hospital, and by overwhelming majorities imposed his will by carrying resolutions in defiance of the Lord Mayor and the Council of the Fund. This led to the formulation of the existing rules of the constitution, which have been modified from time to time, but there is still need for the drawing up of simple rules governing the procedure at the annual meeting of the constituents for the guidance of the Chairman, which should guarantee a fair and uninterrupted discussion each year.

It is perfectly clear the Metropolitan Hospital Sunday Fund cannot continue to prosper or even to endure much longer unless it has behind it the confidence and good will of the clergy and ministers, of the representatives of their congregations, of the contributors and of the public at large. It is therefore a development which should result in great good to the Sunday Fund, that the present Lord Mayor, Sir Thomas Crosbie, has undertaken to go thoroughly into the matters which have led to the existing difference of opinion. We have every confidence that the Lord Mayor will be even better than his promise, and that a small, strong, able and representative committee will be associated with him in considering the whole of the matters involved on their merits. This particular business is one which presses for a prompt solution, and we hope it may be possible for the Lord Mayor to organise the necessary machinery at once, so that all avoidable delays may be prevented.

